# A Ubiquitin Independent Degradation Pathway Utilized by a Hepatitis B Virus Envelope Protein to Limit Antigen Presentation

**DOI:** 10.1371/journal.pone.0024477

**Published:** 2011-09-28

**Authors:** Yuanjie Liu, James S. Testa, Ramila Philip, Timothy M. Block, Anand S. Mehta

**Affiliations:** 1 Department of Microbiology and Immunology, Drexel University College of Medicine, Doylestown, Pennsylvania, United States of America; 2 Immunotope Inc., Doylestown, Pennsylvania, United States of America; Centro Nacional de Microbiología - Instituto de Salud Carlos III, Spain

## Abstract

Hepatitis B virus envelope glycoproteins Large (L), Middle (M) and Small (S) are targets of the host cellular immune system. The extent to which the host recognizes viral antigens presented by infected cells is believed to play a decisive role in determining if an infection will be resolved or become chronic. As with other antigens, HBV envelope polypeptides must be degraded, presumably by cellular proteasomes, to be presented by the MHC I pathway. We have used M as a model to study this process and determine how ER quality control monitors these foreign polymeric proteins and disposes of them through the ER-associated degradation (ERAD) pathway. Using both wild type and mutant HBV M protein, we found that unlike most ERAD substrates, which require ubiquitination for retrotranslocation and degradation, the HBV M protein, which only contains two lysine residues, can undergo rapid and complete, ubiquitin independent, proteasome dependent degradation. The utilization of this pathway had a functional consequence, since proteins degraded through it, were poorly presented via MHC I. To test the hypothesis that the level of ubiquitination, independent of protein degradation, controls the level of antigen presentation, we inserted two additional lysines into both the wild type and mutant M protein. Amazingly, while the addition of the lysine residues dramatically increased the level of ubiquitination, it did not alter the rate of degradation. However and remarkably, the increased ubiquitination was associated with a dramatic increase in the level of antigen presentation. In conclusion, using the HBV surface protein as a model, we have identified a novel ubiquitin independent degradation pathway and determined that this pathway can have implications for antigen presentation and potentially viral pathogenesis.

## Introduction

Newly synthesized secretory and membrane proteins are translocated into the endoplasmic reticulum (ER) co-translationally, where they undergo folding and post-translational modification including N-linked glycosylation before delivery into the secretory pathway [1]–[5]. Proteins that fail to fold correctly or take too long to fold are extracted from the folding cycle by the ER quality control (ERQC) machinery [6]. This cellular function ensures that only polypeptides that attain their native conformation can reach their final destinations and monitors any terminal misfolded proteins to be retrotranslocated out of ER and degraded by the 26S cytosolic proteasome in a series of tightly regulated processes, referred to as the ER-associated degradation (ERAD) pathway [1], [7]. The end result of this pathway is the controlled degradation of misfolded proteins and the generation of peptides for MHC I antigen presentation [8]. Ubiquitination is thought to play an essential role in both the dislocation and proteasomal degradation of misfolded ER associated proteins [9]–[13].

Hepatitis B virus (HBV) specifies three envelope glycoproteins, called large (L), middle (M), and small (S), that are all derived from the same open reading frame (ORF). These proteins are synthesized and translocated into the ER where they undergo folding and potentially N-linked glycosylation before secretion through the trans-Golgi network. The M protein differs from the other HBV envelope proteins in that while the L and S proteins are made and secreted in unglycosylated or N-glycosylated forms [14], the M protein is always secreted as a singly (gp33) or doubly (gp36) glycosylated species. An unglycosylated M species (p30) is found within the cell but the nature and fate of this molecule has, until now, remained a mystery [15].

HBV infection can lead to either an acute or a chronic infection. Whether or not the infection is resolved is believed to depend upon the extent to which host T lymphocytes recognize and clear HBV antigen presenting hepatocytes. To study the pathway of degradation (and presentation) of the HBV envelope proteins, the HBV M protein was employed as a model to study how ER quality control monitors and disposes of secretion incompetent HBV surface proteins through the ERAD pathway. The HBV M protein was chosen as it can be expressed and secreted independently of the other proteins and mutations within it are often associated with pathology [16]–[25]. We found that wild type M protein along with a secretion incompetent M protein mutant [26] could be efficiently retrotranslocated from the ER and degraded via the cytosolic proteasome independent of ubiquitination. Furthermore the utilization of this ubiquitin independent pathway was associated with poor antigen presentation as both wild type and mutant M proteins were poorly presented via MHC I regardless of their rate of proteasomal degradation. Forcing ubiquitination through the addition of two lysine residues resulted in increased antigen presentation without altering the rate of protein degradation. Therefore, using the HBV M protein as a model, a novel ubiquitination independent, proteasome dependent ERAD pathway was discovered. Functionally, a virus such as HBV may utilize this pathway to limit antigen presentation.

## Results

### The HBV M protein does not require ubiquitination for entry into ERAD pathway

**Figure 1:**
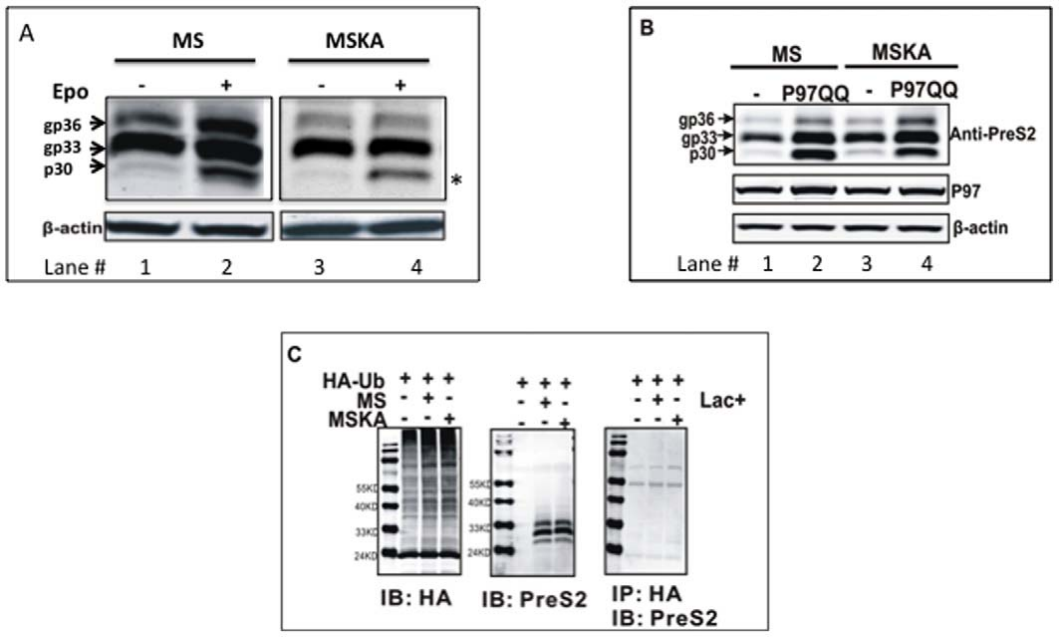
Lysine linked ubiquitination is not required in the proteasome dependent degradation of the HBV M protein. (**A**) In order to test the hypothesis that ubiquitination is not important for the targeting of W/T M protein for proteasome dependent degradation, K141 and K160 on wild type M (MS) was mutated to Alanine (MSKA). The constructs were transfected into 293T cells and either left untreated (-) or treated with proteasome inhibitor epoxomicin (1.5 uM) for 16 h. The cells were then lysed and the intracellular M protein detected via immunoblot using a PreS2 antibody (a.a.13–26 of PreS2). The locations of the three species of wild type M protein are indicated. The asterisk is used to highlight the p30, which accumulate with proteasome inhibition. The level of β-actin was used to monitor loading and is shown below the M immunoblot. (B) The p30, gp33 and gp36 M proteins are ERAD substrates. MS (left) or MSKA (right) were either co-transfected into 293T cells with an empty vector, or a dominant negative P97 (P97QQ) expression vector and the level of M protein (for Pre-S2 domain), p97 and actin measured by immunoblot. Anti-PreS2 immunoblot shows that inhibition of functional P97 by over expression of dominant negative P97 rescued all of the transfected contructs, indicating that p97 is required for the degradation of the HBV M protein in a ubiquitin indepedent manner. (C) Removal of lysine residues indeed blocks ubiquitination of W/T M protein. HEK293T cells were either transfected with HA-Ub alone or cotransfected with MS or MSKA as indicated and left untreated or treated with the proteasome inhibitor Lactacystin (20 uM) for 16 hours. Anti-HA and anti-PreS2 immunoblots were used confirm the expression of each indicated protein. Co-immunoprecipitation was performed using HA affinity gel followed by western blot analysis with anti-PreS2 antibody to detect interaction between HA-tagged ubiquitin and wild type or mutant M proteins. Regardless of the presence of lysine residues (compare MS and MSKA), HA-tagged ubiquitin could not be detected on wild type M protein, confirming the lack of ubiquitin conjugation.

In our previous work examining the folding and degradation of the HBV envelope proteins we noticed that the HBV middle (M), and small (S) envelope glycoproteins, similar to many toxins, have only one or two potential sites of ubiquitin conjugation (lysine residues) [27], [28]. As these toxins can co-opt the ubiquitin-independent retrotranslocation machinery to induce pathology [27], [28], we hypothesized that the removal of misfolded HBV M and S envelope glycoproteins from the ER may occur in a ubiquitin independent manner as well. To test this hypothesis, we examined the degradation of the W/T M protein and a mutant M protein in which the two potential sites of ubiquitination conjugation were altered (K141A and K160A) to prevent lysine mediated ubiquitin conjugation. As shown in Figure 1A, expression of the HBV M protein results in the intracellular accumulation of three bands, which correspond to the unglycosylated or potentially de-glycosylated M protein (p30) [29], a singly glycosylated M protein (gp33) and a doubly glycosylated M protein (gp36). When cells are treated for 16 hours with the proteasome inhibitor epoxomicin, there is primarily an increase in the unglycosylated (p30) form of the M protein (Figure 1A, lane 1 and 2) with smaller increases in the gp33 and gp36 M bands. Similar increases are also seen with full length HBV producing cell lines such as Hep G2 2.215 cells (Figure S1). There are three explanations for this. The first is that the p30 species is an M protein that fails to enter the ER (hence is not glycosylated) and is degraded in a non ER mediated proteasome dependent manner. The second possibility is that the p30 M protein is an unglycosylated M species that fails quality control within the ER and is retrotranslocated into the cytosol for proteasome mediated degradation. The third possibility is that the p30 M isoform is the result of the deglycosylation of the gp33 and gp36 isoforms before degradation by the cytosolic proteasome [29]. That is, the glycans on retrotranslocated glycoproteins are removed in the cytosol by the action of a cytosolic PnGase F before proteasomal degradation [30]. To determine the nature of the p30 species we utilized a dominant negative Cdc48/P97 expression vector to determine the intracellular origin of the p30 M isoform. The cytoplasmic ATPase Cdc48/P97 complex plays a key role in the retrotranslocation of ERAD substrates from the ER through ATP hydrolysis. When this dominant negative variant is expressed, proteins that utilize Cdc48/P97 for retro-translocation will accumulate within the ER [31] before the action of the cytosolic PnGase F. Thus, to determine the nature of the p30 M protein, wild-type M protein was transfected either with a shuttle vector (Figure 1B, Lanes 1) or cotransfected with a dominant negative P97 (P97QQ) construct (Figure 1B, Lane 2) [32]. It is noted that while p97 could be detected in all cells, no increases in total p97 was observed when p97QQ was expressed, which is consistent with other reports using this p97 dominant negative construct [32]. Figure 1B shows that when the function of endogenous P97 was inhibited by P97QQ in cells co-transfected with the M expression vectors, accumulation of the gp36, gp33 and p30 isoforms occurred (Figure 1B, lane 1 and 2). This indicates that all three of these proteins are ERAD substrates and importantly, indicates that the p30 species seen after proteasome inhibition (Figure 1A, lane 2) is the result both of the de-glycosylation of gp33 and gp36 that fails ER quality control and the ERAD of unglycosylated M protein (p30).

Figure 1A also shows that, consistent with the original hypothesis, the alteration of lysines at position 141 and 160 did not result in any substantial increase in any form of the M protein, suggesting that lysine linked ubiquitination is not required for efficient degradation of the M protein (Figure 1A lane 1 and 3). In contrast, similar to what was observed with wild type M protein, treatment with epoxomicin (Figure 1A lanes 3 and 4) lead to an increase in the p30 form of the M protein suggesting that this protein is still degraded via the cytosolic proteasome. In addition, inhibition of ERAD through the use of the p97 dominant negative mutant leads to the accumulation of the same M bands as were observed with the wild type M protein (Figure 1B lane 4, implying that the wild type and lysine negative mutants are degraded in a similar way.

In order to ensure that removing the two-lysine residues completely blocked the ubiquitination of the M protein, a Co-IP was performed to detect whether HA-tagged ubiquitin could be conjugated to either the wild type or the lysine-free mutant in the presence of the proteasome inhibitor Lactacystin [33]. Results shown in Figure 1C indicate that WT M is not ubiquitinated with the HA-tagged ubiquitin as it cannot be immunoprecipitated using HA affinity gel immunoprecipitation. This is true regardless of whether the lysine residues were present or not (right panel of Figure 1C). Thus in conjunction with the above results, the W/T M protein appears to be efficiently degraded via a ubiquitin independent but proteasome dependent manner.

### Secretion incompetent HBV envelope protein mutants also do not require ubiquitination for proteasome dependent degradation

**Figure 2:**
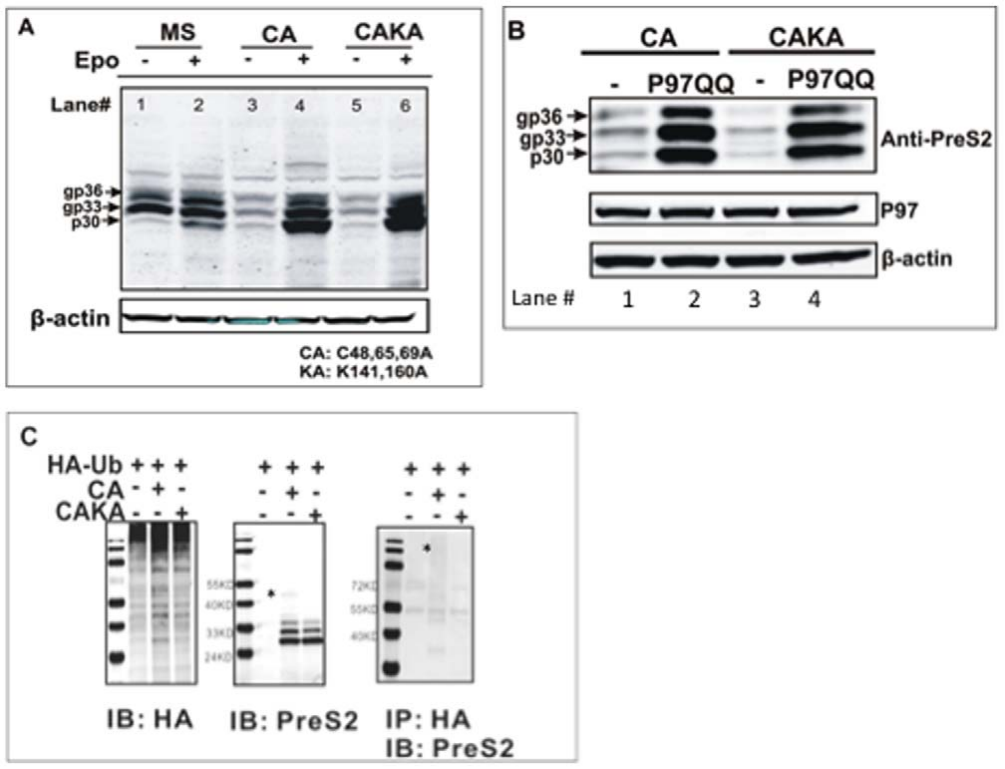
Secretion incompetent M protein is rapidly degraded through an ubiquitination independent, proteasome dependent pathway. (**A**) Cys-48, Cys-65 and Cys-69 on wild type M protein was converted to Alanines, and the construct was named as CA. In order to study the role of ubiquitination in the rapid degradation of the CA mutant protein, all the lysine residues (K141, K160) were mutated to Alanines, and the construct was named as CAKA. Wild type M construct (MS), CA and CAKA were transfected into 293T cells and either left untreated (-) or treated with epoxomicin for 16 hours one day post-transfection. The accumulation of CA mutant protein in the presence of the proteasome inhibitor implies that this protein is being degraded in a proteasome dependent manner; however this degradation process does not require ubiquitination as removal of Lysines did not prevent the rapid degradation by the proteasome. The level of β-actin was used to monitor loading and shown below the M immunoblot (B) The p30, gp33 and gp36 CA M proteins are ERAD substrates. CA and CAKA were either co-transfected into 293T cells with an empty vector, or a dominant negative P97 (P97QQ) expression vector and the level of M protein (for Pre-S2 domain), p97 and actin measured by immunoblot. Anti-PreS2 immunoblot shows that inhibition of functional P97 by over expression of dominant negative P97 rescued all of the transfected contructs, indicating that p97 is required for the degradation of the HBV M protein in a ubiquitin indepedent manner. (C) Removal of lysine residues indeed blocks ubiquitination of CA. HEK293T cells were either transfected with HA-Ub alone or cotransfected with CA or CAKA as indicated, and either left untreated or treated with the proteasome inhibitor Lactacystin (20 uM) for 16 hours. As in Figure 2, anti-HA and anti-PreS2 immunoblots were used to confirm the expression of each indicated protein. However, when lysine residues were removed (CAKA), none of the ubiquitinated misfolded M could be detected as there is no any specific signal comparing to the control lane in which HA-Ub alone was transfected in the cells, indicating that removal of Lysine residues indeed prevents ubiquitination of CA mutant proteins.

Although the results in Figure 1 clearly indicated that the wild type M protein does not require ubiquitination for degradation through the proteasome complex, the overall level of protein degradation was limited. Thus we examined a secretion incompetent M (and S) protein mutant [26] in an effort to determine if a more unstable protein could also utilize this pathway. Cysteine residues C48, 65 and 69, which are all involved in M protein inter-chain disulfide bond formation [26], were changed to alanines, and the expression construct was named as CA. The CA M expressing construct was transfected into 293T cells and the rate of CA M protein degradation was determined by treatment with the proteasome inhibitor epoxomicin. As shown in Figure 2A, the steady state levels of the CA M protein were much lower than wild type unless epoxomicin was included in the cell culture medium, suggesting that CA M protein is rapidly degraded by the cytosolic proteasome. The CAKA mutant, made by changing all the lysine residues in CA to alanines, was also rapidly degraded by the proteasome (compare lanes 5 and 6). Indeed, the levels of the CA and CAKA M protein were identical, regardless of the presence of lysine residues and presumably ubiquitin conjugation (compare lanes 3 and 5). Similar to the wild type M protein that failed ER quality control, Figure 2B shows that when the function of endogenous P97 was inhibited by overexpressing the dominant negative form of P97 (P97QQ), the CA (Figure 2B) mutant dramatically accumulated, as compared with the cells in which only functional P97 (wild-type P97) was present. Similar to the results in Figure 1B, all three CA M isoforms accumulate again implying that these isoforms fail ER quality control (similar to the wild type) and are sent out of the ER for degradation by the cytosolic proteasome. The overexpression of P97QQ could also rescue the nonubiquitinated CA M mutant substrates (CAKA) suggesting that both the wild-type M protein and the CA mutant can enter an ubiquitination-independent, P97-dependent, proteasome-dependent ERAD degradation pathway.

In order to ensure that removing the two-lysine residues completely blocked the ubiquitination of CA, a Co-IP was performed to detect whether HA-tagged ubiquitin could be conjugated to the lysine-free CAKA mutant in the presence of the proteasome inhibitor Lactacystin. In the absence of proteasome inhibition, very limited CA M protein is observed (see figure 2A), hence proteasome inhibition was used to allow for accumulation of protein. Thus the CA M protein observed in the middle panel of Figure 2C represents that CA M protein which is to be degraded by the proteasome. While the results shown in Figure 1C indicate that WT M is not ubiquitinated, a small level of HA-tagged ubiquitin was found to be associated with the CA M protein (Figure 2C). That is, in the CA M protein that contains lysine residues, low levels of HA-tagged polyubiquitinated CA M protein could be detected as a ladder (smear) that was present in cells producing both HA-tagged ubiquitin and the HBV CA protein, which was not present in cells producing just HA tagged ubiquitin (Figure 2C). It is noted that the level is low and much lower than what is observed with other ubiquitinated proteins (shown below). In addition, no evidence of ubiquitination was observed on the CAKA M mutant (Figure 2C) indicates that ubiquitin conjugation was not required for the efficient degradation of this secretion incompetent misfolded M protein. Inhibitors of lysosomal degradation had no impact upon the levels of either wild type or CA mutant, indicating no role for non proteasomal pathways in the degradation of the HBV M protein (Figure S2).

### Role of N-linked glycan in the degradation of HBV envelope proteins

The ERAD pathway involves a set of lectin-like proteins that are involved in substrate recognition and targeting of misfolded protein for ERAD [34]. The ER degradation-enhancing α-mannosidase-like protein (EDEM) is thought to associate with misfolded glycoproteins that have failed the deglycosylation–reglycosylation cycle and have been processed by ER mannosidase I. Similarly, the mammalian homologues of yeast osteosarcoma 9 (OS-9) are thought to bind to glycoproteins processed by the ER mannosidase I and contain terminal α1, 6-linked mannose residues.

**Figure 3:**
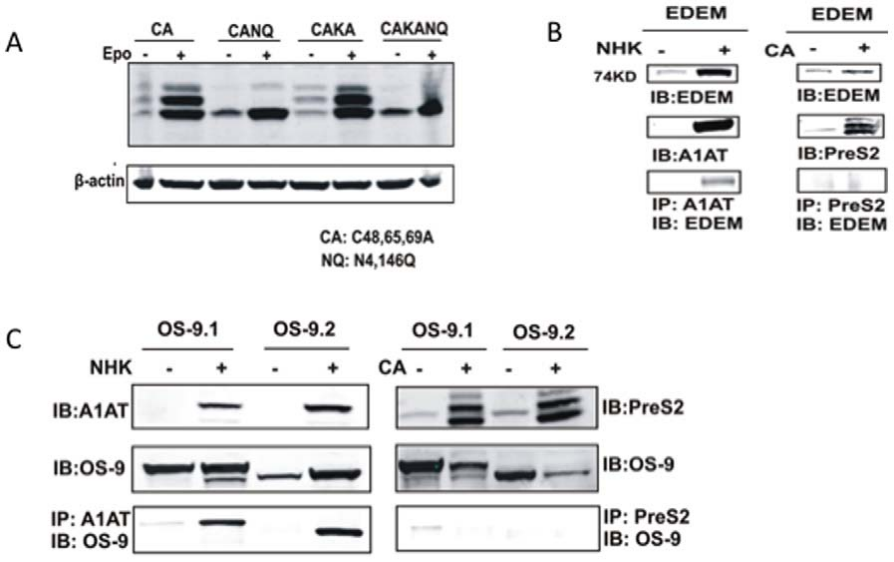
N-linked glycans and the lectin like proteins EDEM and OS-9 are not involved in the degradation of HBV envelope proteins. (A) Removal of N-linked glycan sites on CA or CAKA does not affect the degradation of the M protein. (B) The CA mutant does not interact with EDEM. Either the NHK or CA mutant was transfected alone or together with an EDEM over-expresser into HEK-293 cells and the association between NHK and EDEM or CA and EDEM determined by either immunoprecipitation of NHK or M protein followed by western blot for EDEM. As this Figure shows, while EDEM can be immunoprecipitated with NHK, CA can not. (C) The CA mutant does not interact with OS-9.1 or OS-9.2. Similar to the experiments with EDEM, either the NHK or CA mutant was transfected alone or together with a OS-9.1 or OS-9.2 over-expresser into HEK-293 cells and the association between NHK and OS-9 or CA and OS-9 determined by either immunoprecipitation of NHK or M protein followed by western blot for OS-9.

The role of EDEM and OS-9 was tested either by removing the two (ASN-4 and ASN-146) potential sites of glycosylation on the CA mutant (Figure 3A) or by coimmunoprecipitation studies with EDEM (Figure 3B) or OS-9 (Figure 3C) overexpression vectors. As Figure 3A shows, removal of all glycosylation sites on either the CA mutant or the CAKA mutant had no impact on the level of degradation, implying that the glycans do not play a role in the degradation of these proteins.

We further examined whether EDEM and OS-9 were associated in the degradation of these proteins following cotransfection with the CA mutant. As a control, the null Hong Kong variant of α1-antitrypsin (NHK) was used because it was previously shown to interact with both EDEM and OS-9 [35]. Figure 3B shows that although NHK could be coprecipitated with EDEM, no interaction was observed with the CA mutant, indicating that the M protein is degraded in an EDEM-independent manner.

The role of two major isoforms of OS-9 was examined in a way similar as to EDEM. As Figure 3C shows, although the NHK variant was able to interact with both OS-9.1 and OS-9.2, no interaction was observed with the CA mutant, suggesting no role for these chaperone-like proteins, and potentially the N-linked glycans, in the recognition and degradation of the HBV envelope proteins.

### Ubiquitination, independent of protein degradation, is essential for efficient antigen presentation of the HBV envelope proteins

**Figure 4:**
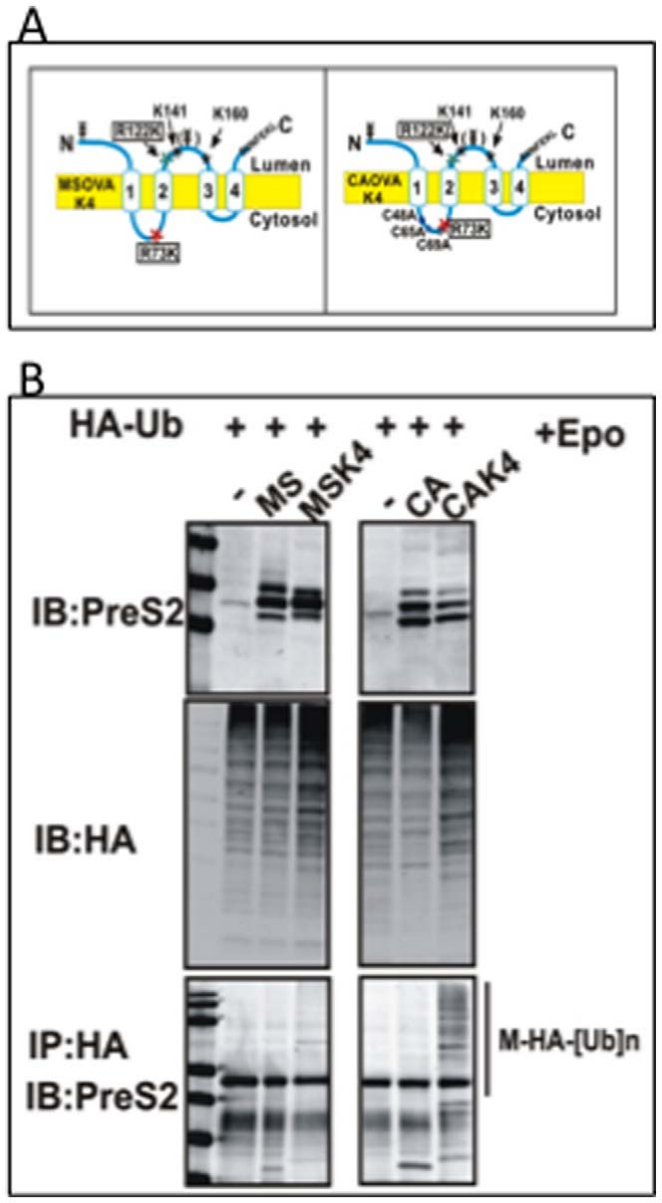
The introduction of two additional lysine residues into the M protein increases the levels of ubiquitination. (A) Two additional lysine residues were introduced at positions 73 (R to K) and at position 122 (R to K) in both the wild type and CA mutants. Constructs with additional lysine residues are referred to as MSK4 and CAK4. (B) Evidence for enhanced ubiquitination was determined using the HA tagged ubiquitin construct as in Figures 1 and 2. Briefly, HEK293T cells were either transfected with HA-Ub alone or cotransfected with MS, MSK4, CA or CAK4 constructs as indicated and either left untreated or treated with the proteasome inhibitor Lactacystin (20 uM) for 16 hours. Anti-HA and anti-PreS2 immunoblots were used to confirm the expression of each indicated protein. Co-immunoprecipitation was performed using HA affinity gel followed by western blot analysis with anti-PreS2 antibody to detect interaction between HA-tagged ubiquitin and wild type or mutant M proteins. While no ubiquitination can be observed with the MS or CA mutants, increased ubiquitination can be observed with the MSK4 CAK4 constructs (a ladder pattern consistent with multi ubiquitination).

Ubiquitination is often associated with enhanced protein degradation and subsequent antigen presentation via the MHC I pathway [8], [36], [37] and a recent report has indicated that ubiquitination was essential for presentation of ER associated antigens [38]. As we did not observe substantial ubiquitin dependent degradation of the HBV envelope proteins, it was of interest to determine if the utilization of this pathway affected antigen presentation. To determine this, we examined the level of antigen presentation of the W/T and CA mutant using an in vitro antigen presentation system. In addition, we modified both the W/T and CA mutant to contain two additional lysine residues, in an effort to determine if we could get increased levels of ubiquitination and determine its effect on antigen presentation. Specifically, arginines at positions 73 and 122 were converted to lysines as these positions contain lysines in other HBV genotypes (as opposed to lysines at position 141 and 160) and these two mutants are referred to as MSK4 and CAK4 (Figure 4A). As an initial test, we determined if ubiquitination could be observed on the MSK4 or the CAK4 mutant. This was done using the same method as in Figures 1C and 2C. As Figure 4B shows, while ubiquitination could not be observed on the MS, or CA constructs, ubiquitination, in the form of a ladder were observed on the MSK4 and CAK4 proteins (Figure 4B, bottom panel). The CAK4 had dramatically increased levels of ubiquitination, presumably as a result of the greater rate of degradation of this protein.

**Figure 5:**
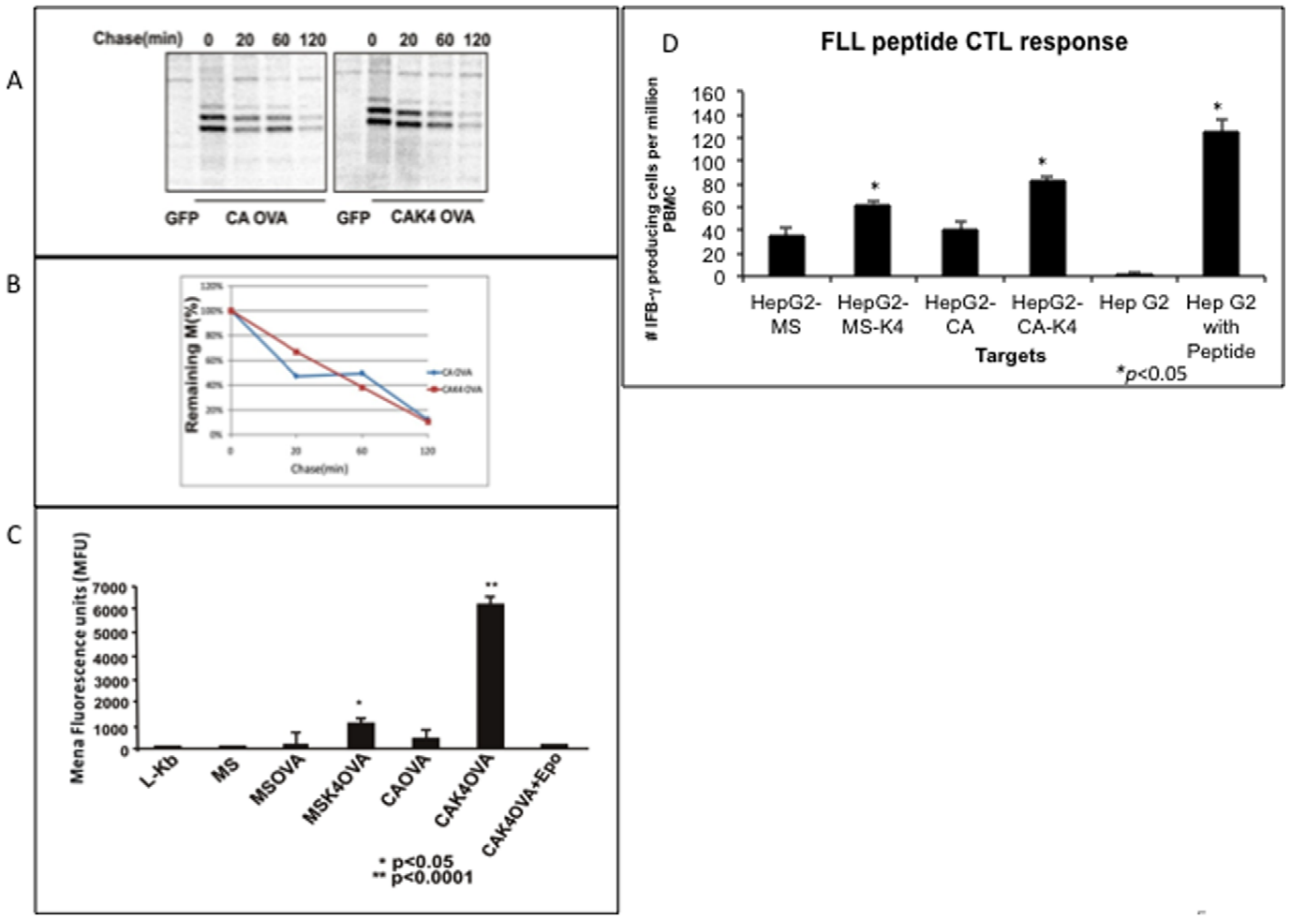
Ubiquitination is required for the efficient presentation of the HBV M protein. (A) Ubiquitination does not alter the rate of degradation of the CA mutant. HepG2 cells transfected with the CA or CAK4 expression construct were pulsed with ^35^S -Met/Cys for 40 minutes followed by 0, 20, 60 or 120 minute chase period. M-specific polypeptides were immunoprecipitated using an antibody to amino acids 13–26 of the pre-S2 domain. The location of the major M protein species is indicated. (B) Quantification of the level of M protein as a function of time of the CA or CAK4 mutant. (C) An in vitro antigen presentation assay highlights the importance of ubiquitination for presentation of the HBV envelope proteins. Briefly, the MS, MSK4, CA and CAK4 expression vectors were engineered to contain the H-2 Kb-restricted OVA257-264 SIINFEKL epitope at the C-terminus of the M protein. Transfection of the MS (non OVA tagged) does not activate the B3.Z CTL clone and hence they produce background levels of luminescence, similar to mock transfected L-Kb cells. In contrast both the MS OVA and the CA OVA M construct lead to the activation of the B3.Z clone. However both the MSK4 OVA and CAK4 OVA constructs lead to statistically significant (p<0.05 for MSK4 and P<0.0001 for CAK4) increases in the level of CTL activation, as compared to the MS or CA Ova constructs. (D) PBMCs isolated from healthy HLA-A2+ human donor blood were stimulated in vitro with peptides corresponding to the HLA-A2–restricted CTL epitope from HBs (FLLTRILTI) The ability of in vitro–generated CTLs to recognize and secrete interferon-γ (IFNγ) was evaluated by ELI-Spot assay. The number of IFN producing cells reactive to MS, MSK4, CA or CAK4 target cells is given. Again both the increase in the MSK4 and CAK4 were statistically significant (P<0.05). Target cells (Hep G2 cells) either mock treated or pulsed with peptide were utilized as the negative or positive controls.

Ubiquitination is thought to enhance antigen presentation through increased proteasomal degradation [39], [40]. The relative stability of CAK4 was compared to the parental CA mutant following transfection into Hep G2 cells and the level of the M protein found in cell lysates determined following a 40 minute pulse with ^35^S methionine/cysteine and either a 0, 20, 60 or 120 minute chase. As Figure 5A shows, after a 40 minute label and 20 minute chase, the level of intracellular M protein was reduced by 50% in both the CA mutant and the CAK4 mutant. The level of this protein continued to decline and was reduced by almost 90% at the 120-minute time point. Importantly, the rate of degradation of the two mutants was equal (Figure 5B).

We next set out to examine the role of ubiquitination in HBV M protein class I antigen presentation. The wild type (MS and MSK4) and CA (CA and CAK4) expression vectors were engineered to contain the H-2K^b^-restricted OVA257-264 SIINFEKL epitope at the C- terminus of the M protein (in the S domain) [41]–[43]. This class I epitope is a useful tool to measure antigen presentation and is engineered with flanking sequences to allow for proper processing and the measurement of antigen presentation [38], . These vectors were transfected into mouse L-K^b^ cells and the level of antigen presentation determined using a CTL clone (B3.Z) for the SIINFEKL epitope [42], [43]. As Figure 5C shows, transfection of the MS expression vector (lacking the SIINFEKL sequence) does not activate the B3.Z CTL clone and has background levels of luminescence, similar to mock transfected L-K^b^ cells. In contrast both the constructs (MSOVA and CAOVA) containing the OVA sequence lead to the low level of activation of the B3.Z clone, suggesting limited levels of antigen presentation. The level of B3.Z activation (as compared to MS) was not statistically significant for MSOVA (p = 0.46) but was for CAOVA (*p*<0.05). However, as shown in Figure 5C both constructs with the additional lysine residues and enhanced levels of ubiquitination (MSK4-OVA and CAK4-OVA) had dramatic increases in the level of OVA peptide presented as compared to wild type MS-OVA or CA-OVA. The MSK4OVA vector lead to a 4.8 fold increase in B3.Z activation as compared to MSOVA, while CAK4OVA lead to an 8.6 fold increase (as compared to CAOVA) (*p*<0.0001). More importantly, this increased level of B3.Z activation correlated with the level of ubiquitination observed in Figure 3. That is, the CAK4 construct, which had dramatically increased levels of ubiquitination (Figure 3B), had the greatest level of antigen presentation. Furthermore, this presentation was proteasome dependent as inhibitors of the proteasome prevented all antigen presentation (Figure 5C).

To ensure that the results obtained with the OVA tagged M protein could also be observed with natural HBV epitopes, we performed a CTL stimulation assay using HBV specific T-cells and HepG2 cells transfected with the above four HBV expression vectors [45]. Activation of HBV specific T cells was measured using an interferon gamma ELI-SPOT assay [45]. As Figure 5D shows, while weak activation could be observed from the MS and CA targets, the MSK4 and the CAK4 constructs show dramatically increased levels (*p*<0.05) of T-cell stimulation, similar to what was observed with the OVA tagged constructs. This result further supports the hypothesis that proteasomal mediated degradation along with ubiquitination is required for efficient presentation of M protein following ERAD.

## Discussion

To study how the cellular ERAD machinery recognizes and disposes of aberrant viral envelope proteins, we created a model system using wild type and secretion incompetent HBV envelope proteins. In our study we found that surprisingly, ubiquitination was not an absolute requirement for the degradation process of either wild type or mutant HBV envelope proteins. Our results indicate that in cell culture, misfolded mutant forms of HBV envelope proteins are effectively removed from the ER and degraded via the proteasome in a ubiquitin independent manner. This represents the first report of a molecule that is removed from the ER and degraded by the proteasome in a ubiquitin independent manner.

Importantly, there is a functional consequence to the utilization of this ubiquitin independent degradation pathway as both the wild type and mutant HBV M proteins were associated with limited MHC I antigen presentation. This was even true of M protein (the CA mutant) that was highly unstable and was rapidly degraded through a proteasome dependent manner. Indeed the wild type M protein and the CA mutant, although they had dramatically different rates of proteasomal degradation, had the same low level of antigen presentation in two different assays (Figure 5). A previous report has indicted that the woodchuck hepatitis B virus pre-S2 protein (WHBV M protein) could prevent the MHC I presentation through a reduction of cell surface MHC molecules [46]. Thus, an attractive hypothesis is that when the M protein is degraded in a ubiquitin independent manner, it takes MHC I with it to the proteasome and thus leads to the reduction of antigen presentation. This was briefly tested by the direct addition of SIINFEKL peptide to M expressing cells and testing for B3.Z activity. In such an assay, there was no major difference in activity between M expressing cells and mock transfected cells. However, more sensitive assays are required to truly determine the role of M on the MHC I.

The addition of two extra lysine residues, which resulted in increased levels of ubiquitination, resulted in increased levels of antigen presentation for both the wild type, and more impressively for the CA mutant. Importantly the increase in ubiquitination did not result in any change in the rate of degradation. Therefore it appears that ubiquitination, independent of protein degradation, is important for the efficient presentation of the HBV M and S envelope proteins. In addition, by limiting the number of lysine residues on the M and the S proteins, the virus theoretically can limit the presentation of peptides from these highly abundant proteins thus providing a selective advantage for the virus. It is noted that the HBV L protein contains two additional lysine residues in the Pre-S1 domain and its degradation pathway may be fundamentally different than that observed with the M protein. Indeed the expression of the HBV L protein is thought to occur latter during the natural infection (after the other envelop proteins) to allow for CCC DNA formation through recycling of the rcDNA back to the nucleus [47]–[50]. The level of the L protein controls this cycle, as it is essential for secretion of enveloped virus.

Recent reports have indicated that the 20S proteasome has been known to degrade non ubiquitinated proteins (highly inefficiently) and it is possible that this could impact antigen presentation [51]–[53]. Indeed, it has even been suggested that the 20S proteasome may present peptides from rapidly degraded proteins [51]. However, degradation through the 20S proteasome should still lead to the production of the SIINFEKL epitope [8], which was not observed in our case.

It is noted that glycoproteins with low lysine contents can become ubiquitinated on non lysine residues and thus be efficiently removed and degraded through ERAD [54]. However, in our case, when we examined total protein linked ubiquitin conjugation, it was not observed suggesting that these protein may undergo ubiquitin independent ERAD.

Thus, this report may represent one of the few examples of a protein that could be degraded in a ubiquitin independent but proteasome dependent ERAD pathway [53]. That is, most polytopic membrane-anchored ERAD substrates, such as the Cystic Fibrosis Transmembrane Conductance Regulator (CFTR) protein require ubiquitination catalyzed by the ER membrane associated E3 ubiquitin ligase. This polyubiquitination has been seen as an essential step in the retrotranslocation of proteins out of the ER for degradation by the cytosolic proteasome [55], [56].

While polyubiquitination of ERAD substrates seems to be the general rule, there are several examples of ubiquitin independent dislocation in ERAD. Yu et al reported in 1997 that removing all the lysine residues on TCR-α did not change it's degradation kinetics, suggesting that ubiquitination was not required for degradation of TCR-α [57]. However, this study could not provide definitive evidence that TCR-α mutant completely lacked ubiquitination. Expanding upon these initial results, Yu and Kopito in 1999, inhibited the cellular ubiquitination system, and found that degradation of TCR-α and KαR was suppressed indicating that ubiquitination was indeed required for the dislocation and degradation of TCR-α [12]. Cholera toxin A1, a non-ubiquitinated substrate, utilizes a retrotranslocation process from the ER into the cytosol in order to induce toxicity by activating cytosolic cAMP. However, Cholera toxin A1 is not a typical ERAD substrate and unlike other misfolded ER proteins and the results presented here, can escape degradation after a P97 independent dislocation event [32]. In addition, the HCV F protein has been shown to be degraded in an ubiquitin independent manner [53]. Interestingly, while this protein has been shown to associate with the ER, its proteasomal degradation is mediated through direct interaction with the proteasome α3 subunit.

A remaining question is how the cellular ERAD system recognizes and dislocates the two forms of misfolded M protein. Our study shows here that both wild type and misfolded M protein undergo P97 dependent dislocation and proteasome dependent degradation. Since Derlin-1, the human homologue of yeast Der1p, has been shown to be involved in the degradation of other ERAD substrates through association with P97 to form a channel like structure facilitating the dislocation process [58], [59], it might be a key factor involved in the degradation of misfolded M protein. ER chaperones, including BiP, calnexin (CNX), and calreticulin (CRT) promote folding of ER proteins. However, unsuccessful cycles of folding or assembly may target these proteins for retrotranslocation [60]. EDEM accelerates ERAD by extracting immature monoglucosylated proteins from the CNX-CRT cycle in a glycan dependent manner [61]. However, our work suggest that neither Calnexin nor EDEM play a role in the degradation of the HBV M protein, since the removal of all the N-glycan on M did not alter its degradation by the proteasome (Figure 3).

It is noted that a recent report has highlighted the importance of ubiquitination in the degradation of ER associated antigens as opposed to cytosolic antigens [38]. Our work supports the hypothesis that ubiquitination is essential for ER associated antigens, but differs in our work, ubiquitination did not alter the rate of protein degradation. It is important to note that efficient antigen presentation of the M protein requires both ubiquitination and proteasome mediated degradation as proteasome mediated degradation was not sufficient for antigen presentation (Figure 5).

In conclusion, we report that the HBV envelope protein that fails ER quality control can enter into the ERAD pathway independent of ubiquitination. These proteins are subsequently recognized and efficiently degraded via the cystolic proteasome complex. The HBV envelope proteins represent one the few examples of proteins that can efficiently be removed from the ER and degraded via the proteasome in a ubiquitin independent manner and by doing so, can limit antigen presentation.

## Materials and Methods

### HBV M expression constructs

The HBV middle and small surface protein sequences (nt-3174-1-1980) were derived from subtype ayw (VO1460) through pfu PCR (Stratagene, La Jolla, CA) amplification and cloned into pCMV-Tag4A (Stratagene) plasmids by the removal of FLAG and SV40 polyA sequences from the backbone. This plasmid is referred to as pCMV-MS. The triple cysteine mutant CA was made by mutating the triplet codes (TGTs) of Cysteine residues at positions of 48, 65 and 69 in the S region into GCTs via a joint PCR method, introducing the substitution of Cysteines to Alanines. Mutagenesis of Lysine residues (K141, 160) into Alanines on either the wild type M construct or the CA construct was performed by using the joint PCR method, and the constructs were named MSKA and CAKA, respectively. All the constructs were sequenced for coding fidelity. Using the same mutagenesis strategy, two arginine residues, R73 and R122, were mutated into lysines in both the wild type (MS) and the CA mutant and these vectors are referred to as MSK4 and CAK4. The OVA 257–264 SIINFEKL epitope as placed at the c-terminus of all expression vectors generating the MSOVA, MSK4OVA, CAOVA and CAK4OVA constructs. As we have done before, the SIINFEKL construct was flanked by 4 amino acids on each side to ensure proper processing and presentation. The entire sequence as inserted was as follows: SEQLE-*SIINFEKL* **-**TEWTS.

### OS-9.1, OS-9.2 and EDEM expression vectors

Total cellular RNA from 293T cells was extracted with TRIzol reagent (Invitrogen). First-strand cDNA was synthesized with SuperScript III DNA polymerase (Invitrogen) and an oligo(dT)12–18 primer. Two splicing isoforms OS-9.1 and OS-9.2 were amplified from HEK293 cells by using the following primers: GGG AAC GAA AGA TGG CGG CGG AAA CGC T (forward) and GTT GGT CTC AGA AGT CAA ATT CGT CCA(reverse) [62]. The purified PCR fragments were cloned into pCDNA3.1/V5-His-TOPO expression vector (Invitrogen, Carlsbad, CA) and the cDNAs corresponding to two different splicing isoforms OS-9.1 and OS-9.2 were verified by sequencing. Since one stop codon was engineered in the construct upstream of the coding region of the tags, OS-9 proteins generated from these constructs do not contain C-terminus tags. EDEM gene was released from full length expressed sequence tag(Cat# IHS1380-97431075, Open Biosystems) by SnaBI and NotI digestion and the fragments were cloned into EcoRV and NotI sites on expression vector pCDNA3(Invitrogen, Carlsbad, CA). All the constructs were sequenced for coding fidelity.

### Cell culture and plasmid transfection

293T cells [63] were cultured in 6-well plates (Corning, Corning, NY) with DMEM/F12 medium (Invitrogen, Carlsbad, CA) containing 10% fetal bovine serum at 37°C with 5% CO2. At the time when cells reached 90% confluence, Fugene 6 (Roche, Palo Alto, CA) was used to carry out transfection of plasmid DNA according to manufacturer's instruction. Proteasome inhibitor Lactacystin and epoxomicin (Calbiochem, Gibbstown, NJ) were dissolved in DMSO and added to the culture medium for 16 h treatment one day post-transfection at concentrations of 20 uM and 1.5 uM, respectively.

### Antibodies

Polyclonal rabbit antiserum against the HBV PreS2 domain was purchased from Fitzgerald Industries Intl. (Acton, MA). Anti-HA (clone 16B12) was purchased from Covance Inc. (Princeton, NJ). A mouse anti-actin antibody (Chemicon, Temecula, CA) was used for protein normalization. Rabbit polyclonal anti-VCP (P97) and anti-OS-9 antibodies were purchased from Novus Biologicals, LLC (Littleton, CO). Monoclonal Anti-AAT and rabbit polyclonal anti-EDEM 1 were purchased from Santa Cruz Biotechnology Inc. (Santa Cruz, CA) and Sigma-Aldrich Inc. (Saint Louis, MO) respectively.

### Immunoblotting analysis

Cells in 6-well plates were lysed in 300 µL lysis buffer containing 50 mM Tris-HCl, pH 7.4, 1 mM EDTA, 0.5% NP-40 plus fresh 1× protease inhibitor cocktail (Sigma, St. Louis, MO) and 30 mM N-ethylamaleimide (Sigma). Lysates were denatured in Laemmili buffer and resolved in 12% SDS PAGE gels. For all samples, protein quantification was performed and proteins loaded in equal amounts. Proteins were blotted onto PVDF membrane. After 1 hour blocking, using LI-COR blocking buffer (LI-COR, Lincoln, Nebraska), primary antibody was applied and incubated at room temperature (RT) for two hours. In this study, anti-PreS2 and anti-P97 were diluted at 1∶1000 blocking buffer. After washing 3 times with PBS+0.1%Tween-20, the membrane was incubated at RT for 1 hour in blocking buffer containing 1∶5000 diluted IRDye conjugated secondary antibody (LI-COR) corresponding to the source of primary antibody. After three 5 minute RT washes, the membrane was scanned using the ODYSSEY Infrared Imaging System (Li-Core) and the signal was quantitatively analyzed according to manufacturer's instruction.

### HA tagged polyubiquitination assay

An HA-Tagged ubiquitin system previously reported was utilized to determine the level of protein linked ubiquitin [33]. This system has previously been shown to mimic the level of ubiquitin conjugation. Briefly, 2 ug HA-ubiquitin expression vectors were cotransfected with either 3 µg shuttle vectors or 3 µg HBV M expression vectors into 293T cells in a 60 mm^2^ dish. One day post transfection, the cells were treated with proteasome inhibitor Lactacystin(20 µM) for 16 hours. Thereafter the cells were lysed in 600 µL 30 mM NEM containing lysis buffer, cell debris and nuclei were removed by brief centrifugation at 4°C. 250 µL supernatants diluted with 350 µL binding buffer (50 mM Tris-HCl pH7.4, 150 mM NaCl_2_, 2 mM EDTA, 0.25% NP40 and 2% BSA) were mixed with 20 µl EZ view HA-tagged affinity gel (Sigma). After 2 hours rocking at 4°C, the beads were precipitated and washed 3 times with 800 µl washing buffer of 50 mM Tris-HCl, pH 7.4, 150 mM NaCl_2_ (500 mM NaCl_2_ for the last wash), 5 mM EDTA and 0.5% NP40. The pellet was desalted with one additional wash with 20 mM Tris-HCl, pH 7.4 and was denatured in 20 µl 2× Laemilli loading buffer.

### S^35^ labeling and immunoprecipitation

Transfected cells in a 6-well plate were washed with pre-warmed PBS once and cultured in 1 ml DMEM labeling medium in absence of methionine and cysteine (Invitrogen) for 30 minutes. The culture medium was then replaced with 1 ml pre-warmed DMEM labeling medium containing 100 uCi S^35^-methionine/S^35^-cysteine (NEN, NEG072 EXPRE35S35S). After 40 minutes pulse, cells were chased with regular culture medium at various time points. Labeled cells were lysed on ice for 30 minutes in 750 ul pre-cold RIPA buffer (50 mM Tris-HCl, pH7.4, 150 mM NaCl_2_, 1% Triton X-100, 0.5% sodium deoxycholate, 0.1% SDS and 1× protease inhibitor). Cell debris and nuclei were removed by brief centrifugation at 4°C and the supernatants were transferred into a new tube containing 20 µL anti-PreS2 antibody coated Protein A/G agarose beads (Santa Cruz). After 2 hours rocking at 4°C, the beads were precipitated and washed 3 times with 800 uL washing buffer of 50 mM Tris-HCl, pH 7.4, 150 mM NaCl_2_ (500 mM NaCl_2_ for the last wash), 5 mM EDTA and 0.5% NP40. The pellet was desalted with one additional wash with 20 mM Tris-HCl, pH 7.4 and was denatured in 20 ul 2× Laemilli loading buffer. After electrophoresis in 12% SDS PAGE, the gel was transferred on PVDF membrane, which was exposed and quantified by phosphor-image (Bio-Rad, San Francisco, CA).

### SIINFEKL Antigen presentation assay

The L-Kb cells were cultured in a 24-well plate. The W/T MS, MS K4, CA and CAK4 vectors were transfected into mouse L-Kb cells using with Lipofectamine transfection reagent (Invitrogen) according to the manufacturer's directions. After 1 day, the T cell hybridomas (B3.Z) were added and incubated overnight [44]. The cells were collected and lysed in 100 µl cell lysis solution containing 1× protease inhibitors. 5 µl cell lysates were used for a chemiluminescent reporter assay to measure the level of β-galactosidase produced by the B3.Z clone. The β-galactosidase gene allows for easy monitoring of CTL recognition and activation and the Galacto-Light Plus purchased from Tropix (Bedford, MA).

### In Vitro Generation of Peptide-Specific CTLs

Heparinized blood from healthy human leukocyte antigen A2 (HLA-A2) donors was purchased from Research Blood Components, LLC (Brighton, MA). Peripheral blood mononuclear cells (PBMCs) were purified and cultured as described [64], [65]. After initial stimulation with the synthetic HBV peptide FLLTRILTI, which has been shown to be the major HBV epitope [64], [65], T cells were restimulated with CD4/CD8 T cell–depleted autologous monocytes pulsed with synthetic peptide at 10 µg/mL for 5 days. Interleukin-2 treatment and in vitro restimulation were repeated thrice prior to use of in vitro expanded T cells in enzyme-linked immunosorbent spot (ELISpot) assays. Our previous work has demonstrated that T cells expanded in this manner secrete granzyme B and have surface CD8, hallmarks of the cytolytic potential of CD8+ T cells, so we refer to these cells as CTLs [65].

### ELISpot Assays

In vitro–expanded CTLs were used as effectors in ELISpot assays to assess antigen-stimulated interferon-c release according to the manufacturer's instructions (BD-Pharmingen, San Jose, CA). Target cells were HepG2 human hepatoma cells (HBV negative; American Type Culture Collection, Manassas, VA) transfected with 1 µg of either the MS, MSK4, CA, or CAK4 expression vector as targets in ELISpot assays, and were washed before incubation with T cells. Typically, 2×10^5^ effectors (T cells) and 5×10^3^ targets were used (40∶1). Results are presented as number of interferon γ producing cells per 10^6^ CD8+ T cells.

## Supporting Information

Figure S1 **Inhibition of the cytosolic proteasome leads to the accumulation the HBV p30 M protein species in HBV producing cell lines.** Hep G2.2.15 cells were treated for 16 hours with 1.5 uM epoxomicin and cell lysates examined using an anti-pre-S2 antibody. As this figure shows, when the proteasome is inhibited there is an accumulation of several M protein species, including the p30, gp33 and gp36 forms. As the HBV L protein also contains the pre-S2 domain, accumulation in the L protein are also observed following proteasome inhibition.
(TIFF)

Figure S2 **HBV M proteins are degraded in a lysosome-independent but proteasome-dependent manner.** In order to identify the intracellular proteolytic compartments mediating the degradation of HBV M protein, the pharmacologic inhibitors chloroquine and epoxomicin, which specifically inhibit the function of the lysosome and proteasome respectively, were applied in the medium at the indicated concentration one day post-transfection. After the overnight treatment, the cells were lysed and subjected to the western blot using an anti-preS2 antibody. The accumulation of wild type M or the CA mutant protein occurred in the presence of the proteasome inhibitor only and implies that the HBV M protein is degraded in a lysosome independent, but proteasome dependent manner.
(TIFF)
