# Supplementary figures and images for: A Ubiquitin Independent Degradation Pathway Utilized by a Hepatitis B Virus Envelope Protein to Limit Antigen Presentation
Source: PLoS One. 2011 Sep 28;6(9):e24477. doi: 10.1371/journal.pone.0024477 (PMC3182176; doi:10.1371/journal.pone.0024477)

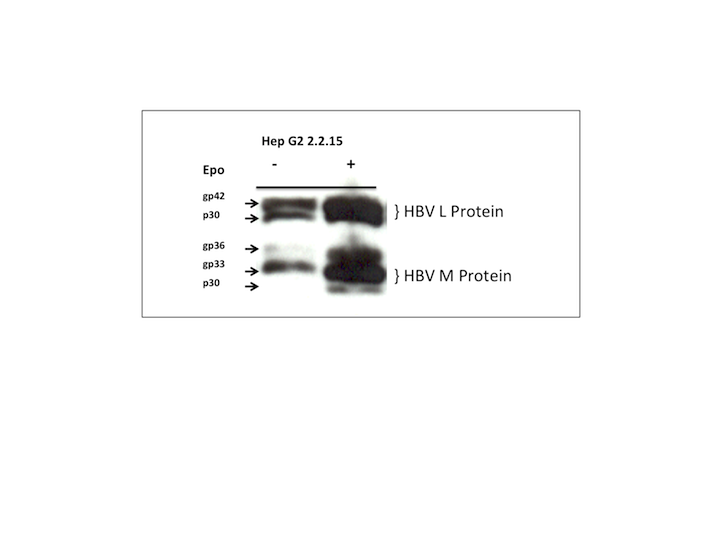

Supplement: Figure S1 — Inhibition of the cytosolic proteasome leads to the accumulation the HBV p30 M protein species in HBV producing cell lines. Hep G2.2.15 cells were treated for 16 hours with 1.5 uM epoxomicin and cell lysates examined using an anti-pre-S2 antibody. As this figure shows, when the proteasome is inhibited there is an accumulation of several M protein species, including the p30, gp33 and gp36 forms. As the HBV L protein also contains the pre-S2 domain, accumulation in the L protein are also observed following proteasome inhibition. (TIFF) [file pone.0024477.s001.tif]

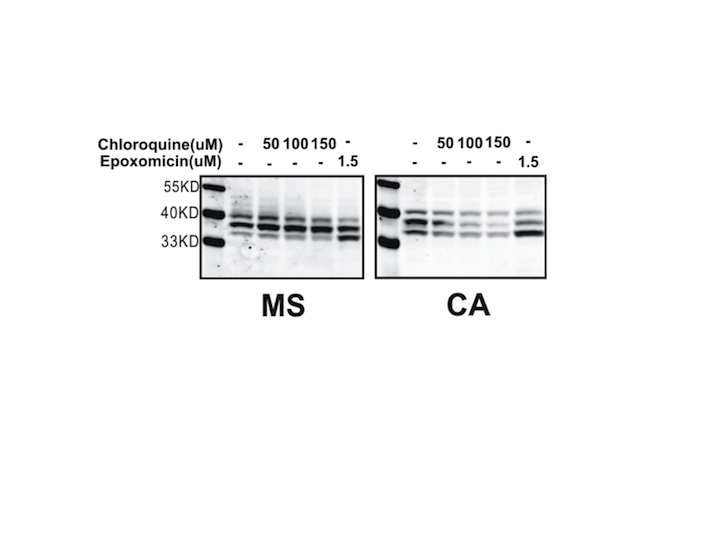

Supplement: Figure S2 — HBV M proteins are degraded in a lysosome-independent but proteasome-dependent manner. In order to identify the intracellular proteolytic compartments mediating the degradation of HBV M protein, the pharmacologic inhibitors chloroquine and epoxomicin, which specifically inhibit the function of the lysosome and proteasome respectively, were applied in the medium at the indicated concentration one day post-transfection. After the overnight treatment, the cells were lysed and subjected to the western blot using an anti-preS2 antibody. The accumulation of wild type M or the CA mutant protein occurred in the presence of the proteasome inhibitor only and implies that the HBV M protein is degraded in a lysosome independent, but proteasome dependent manner. (TIFF) [file pone.0024477.s002.tif]
